# Real-World Results from Combined Screening for Monogenic Genomic Health Risks and Reproductive Risks in 300 Adults

**DOI:** 10.3390/jpm12121962

**Published:** 2022-11-28

**Authors:** Robert S. Wildin, Diana L. Gerrard, Debra G. B. Leonard

**Affiliations:** 1 Laboratory Medicine and Pediatrics & Departments of Pathology, Robert Larner M.D. College of Medicine at the University of Vermont, University of Vermont Health Network, Burlington, VT 05401, USA; 2 Laboratory Medicine & Department of Pathology, University of Vermont Medical Center, Burlington, VT 05401, USA; 3 Laboratory Medicine & Department of Pathology, Robert Larner M.D. College of Medicine at the University of Vermont, University of Vermont Health Network, Burlington, VT 05401, USA

**Keywords:** population health, clinical genomics, primary care providers, genomic screening, gene panels, real-world experience, inherited health risk, carrier status

## Abstract

New methods and demonstrations of feasibility guide future implementation of genomic population health screening programs. This is the first report of genomic population screening in a primary care, non-research setting using existing large carrier and health risk gene sequencing panels combined into one 432-gene test that is offered to adults of any health status. This report summarizes basic demographic data and analyses patterns of pathogenic and likely pathogenic genetic findings for the first 300 individuals tested in this real-world scenario. We devised a classification system for gene results to facilitate clear message development for our Genomic Medicine Action Plan messaging tool used to summarize and activate results for patients and primary care providers. Potential genetic health risks of various magnitudes for a broad range of disorders were identified in 16% to 34% of tested individuals. The frequency depends on criteria used for the type and penetrance of risk. 86% of individuals are carriers for one or more recessive diseases. Detecting, reporting, and guiding response to diverse genetic health risks and recessive carrier states in a single primary care genomic screening test appears feasible and effective. This is an important step toward exploring an exome or genome sequence as a multi-purpose clinical screening tool.

## 1. Introduction

Disease screening, including genetic and genomic screening, is standard of care for many areas of clinical practice. Screening women and their reproductive partners for recessive genetic disease carrier status is a longstanding medical practice in pre-conception and prenatal settings [1,2]. The goal is to identify and offer alternative reproductive options and specialty obstetrics care for couples with a high risk of having children with primarily childhood onset diseases with strong health and longevity impacts. Population-wide screening of individuals for genetically influenced health conditions or increased risk markers is also typical medical practice and is predicated on the availability of care pathways that can modify the onset, time to detection, disease manifestations, severity, or exposure to other risk factors [3]. Screening of adults for cardiovascular disease, diabetes, metabolic disease precursors, mental health, and several cancer types using non-genetic methods is standard of care, and testing for genetic cancer predisposition upon diagnosis with certain cancers is becoming standard of care [4,5]. The goals of adult screening are to intervene early in disease progression with medical measures, early and more frequent screening tests, and patient awareness and modifying lifestyles through education. Most infants in the United States undergo primary newborn screening using non-nucleic acid analytes [6]. Routine screenings for vision, hearing, development, and growth are standard in pediatric settings and failed screens often trigger genetic evaluations [7]. The evidence supporting improved outcomes for several screening measures have earned ratings by the United States Preventative Services Task Force that facilitate access to screening without financial barriers [8].

Current guidelines for genetic disease prevention favor targeted, multi-modal evaluation of close relatives of individuals already diagnosed with known hereditary conditions. These statistically at-risk individuals are often ascertained by the primary care or other provider taking a family health history [9]. While such ascertainment is effective, it cannot identify all people with health risk-associated genetic variants in the general population because many genetically at-risk individuals lack an affected or diagnosed relative [10]. More than fifty percent of individuals from unselected populations found to bear risk variants do not meet family history-based criteria for targeted genetic screening or know they are at increased genetic risk [11]. Furthermore, uptake of testing among family members who are at risk is incomplete [12,13]. These shortcomings limit the effectiveness and efficiency of prevention paradigms in current use.

The multiplicity of approaches to detect genetic disease risk during health care has solid roots, but each element has limitations. We sought to leverage the falling cost of clinical gene panel tests to bring health risk and carrier status knowledge to a greater number of individuals. We targeted a greater range of genetic conditions to extend the reach of screening for genetic health risks beyond pregnancy-related carrier screening, targeted testing initiated due to diagnosis of symptomatic diseases such as cancer or heart disease, or due to a strong family history. We piloted genomic sequencing of 432 genes for dominant and recessive disorders of clinical interest in any-health-status adults as a means of identifying individuals or couples at risk but not captured by the traditional screening approaches.

We previously reported the design and implementation of this clinical genomic screening pilot in the primary care setting [14]. Here, we report cohort characteristics and the aggregated genomic findings from the first 300 patients. We introduce a clinical gene result classification system to guide messaging of clinical and reproductive implications of results. We discuss these observations in the context of previous studies and opportunities for further innovation in genomic population health. 

## 2. Materials and Methods

### 2.1. Testing Program and Analysis Tools

The implementation design and experience, the gene panels used, the testing and reporting process, and the care pathway program guiding clinicians locally have been previously reported [14]. We used existing clinical gene panel tests from Invitae Corp., the Comprehensive Carrier Screen and the Genetic Health (Pro-active) Screen [15]. Clinical significance of variants was classified according to the Sherloc system [16]. To replicate conditions of Affordable Care Act-qualified screenings, i.e., no financial accessibility barrier, we offer the test and test-related genetic counseling at no cost [14]. 

Result data were tracked in a HIPAA-compliant on-premises data server. A data freeze in June 2022 when results from the 300th tested individual were reported, was extracted and analyzed excluding personally identifiable information. Software used in analysis included Microsoft Excel 2016, Access SQL 2016, Visual Basic 2016, and Visual Basic for Applications 2016. 

This analysis represents a quality management activity and does not constitute research according to the institution’s institutional review board (IRB) protocols. 

### 2.2. Cohort

The first 300 patients with completed test results between 1 November 2019, and 31 May 2022 in our ongoing clinical genomic population health screening program comprise the patient cohort. All patients were asked about interest in testing by their usual primary care team as part of preventive care. Each had the opportunity to review printed educational materials and to ask questions of genetics experts prior to and after testing, and all signed a printed clinical genomic testing informed consent document as a prerequisite to testing.

As previously described, the test eligibility criteria were (a) at least 18 years of age, (b) not pregnant and partner not pregnant at the time of testing, (c) patient is an attributed life under Vermont’s accountable care organization, and (d) patient’s primary care provider had received training from the genomic team in discussing testing, consenting, ordering, returning results delivered in the electronic health record, and managing subsequent care. 

### 2.3. Variant Inclusion

Variants of uncertain significance (VUS) and benign or likely benign variants are not reported. We do not differentiate between variants classified as pathogenic (P) and likely pathogenic (LP) since they differ in the level of evidence of pathogenicity but not in clinical care recommended. When new evidence results in subsequent variant reclassification, the clinical laboratory issues amended reports. Our analysis is based on the variant classifications (“on report” status) as of the freeze date. Reclassifications of variant pathogenicity reported after the freeze date, including those resulting in new gene variants reported (from VUS to P or LP) or ones deleted (P/LP to benign or likely benign) are not reflected in this analysis. This does not materially affect the results or conclusions. 

### 2.4. Gene Result and Clinical Class Definitions

Since individuals may have more than one P and/or LP variant identified in a gene, we defined a “gene result” as the gene and all its reported variants and their zygosity. A gene result differs from a genotype in that sources of uncertainty, such as a lack of allelic phase information and their impact on clinical and reproductive messaging, are specified. 

**Figure 1:**
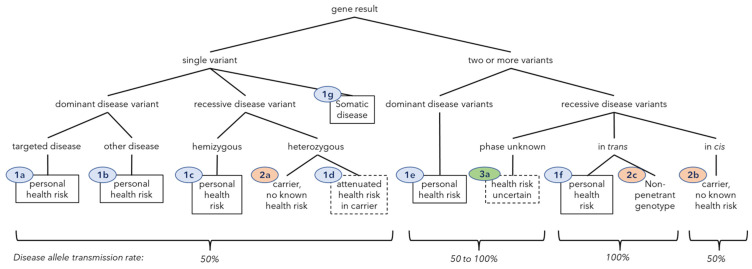
Classification of gene results by number of P/LP variants in a gene, phase, health risk, and inheritance pattern. “Other disease” means a condition other than that typically driving inclusion in screening panels. Ovals contain clinical classes: Class 1 (blue)—personal health risk, Class 2 (orange)—carrier or double carrier only, Class 3 (green)—health risk uncertain. The associated text summarizes the clinical impact. Solid boxes denote a personal health risk that is well-defined; dashed boxes mean attenuated disease risks or technically uncertain risk. Predicted rates of allele transmission to offspring appear at the bottom.

Gene results fall into natural classes with respect to the combination of clinical relevance and risk to offspring for the individual, i.e., the elements upon which genetically informed healthcare activities and options are based (Figure 1). We use the term “clinical classes” to describe these impact groups. 

The classes refer to the clinical importance of the gene result. A Class 1 gene result indicates a potential health risk to the individual who was tested. Class 2 indicates the gene result imparts no known health risk to the tested individual even though they carry one or more P or LP variants; these are predominantly recessive disease carriers. Class 3 indicates uncertainty as to whether the gene result represents a health risk or only a conditional risk due to transmission to offspring. The main Class 3 case is the presence of two or more variants for recessive conditions where the allelic phase was not resolved by the laboratory, resulting in uncertainty as to whether the gene result represents unaffected carrier of a recessive condition (if the variants are in *cis*), or potentially predicted to be affected with a recessive condition (if they are in *trans*).

Class 0 (zero) means no reportable results were identified in any tested gene and refers to individuals, not gene-results. In Class zero, the clinically important information is that known P and LP variants were not identified. The risk profile of the individual is either unchanged or may be lower than average because of testing.

## 3. Results

### 3.1. Cohort Characteristics

**Figure 2:**
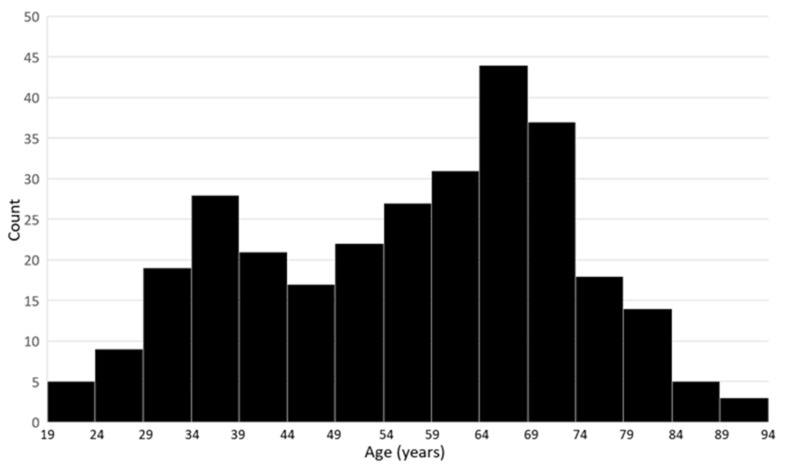
Cohort age distribution histogram (bin size: 5 years).

The cohort shows a slight predominance of women (57%) and a broad distribution of age in years at the time of testing. Minimum and maximum ages were 19 and 92 years, respectively, and a median age of 60 years. Figure 2 is an age histogram showing two age distribution peaks, the larger one in older adults, and a lesser peak in younger adults. Thirty-three percent of tested adults were under age 50 years, meeting the World Health Organization’s definition for reproductive age [17].

### 3.2. Gene and Variant Prevalence

Six hundred and thirty-two gene results were identified in 173 genes among the 300 patients, meaning 0.5% of the 129,600 gene sequencing events yielded a reportable gene result. Three hundred and eight unique P and LP variants were identified. Reportable variants were not identified in 260 of the 432 genes evaluated (Table 1).

### 3.3. Prevalence of Gene Results by Clinical Class

Testing returned no reportable P or LP variants, i.e., Class zero, in 33 individuals (11% of the cohort). The remaining 267 individuals (89%) have at least one P or LP gene variant found. To further understand the personal health risks to tested individuals and the risks to their children, all gene variants were grouped in “clinical classes” (Figure 1) and the numbers of individuals with variants in each class were tabulated (Table 2). All gene results, grouped by clinical class, are presented in Supplementary Materials, Table S1.

#### 3.3.1. Class 1—Predicted Health Risk

Class 1 encompasses gene results associated with an increased risk for a specific health condition in the tested individual. Class 1a connotes a heterozygous gene result associated with a dominant or co-dominant condition that is the main reason that gene is included in the screening panel. Among the cohort of three hundred individuals, 47 (15.7%) individuals have at least one Class 1a dominant, heterozygous, disease or risk-associated gene result. Of 632 total gene results, 51 (8.1%) were Class 1a gene results. A small number of individuals have more than one Class 1a gene result.

Class 1b connotes a heterozygous result associated with a dominant or co-dominant condition different from that typically cited as the screening target for that gene. The single Class 1b gene result involved the *APOB* gene and predicts Hypobetalipoproteinemia rather than Familial Hypercholesterolemia.

Clear or suspected mosaicism for a Class 1a or 1b gene result is specifically annotated as such, but not reclassified. One mosaic variant in *NF1* and one possibly mosaic variant in *TP53* were reported in different individuals. Thus, of the 632 gene results, two (about 0.3%) are mosaic dominant health risk results.

Class 1c is used for a single recessive variant or haplotype that predicts a health risk because there is no wild-type gene copy present. Individuals having a single X chromosome and who are hemizygous for an X-linked recessive variant comprise this group and are expected to be affected by the associated disorder or express the associated susceptibility to illness. The same Class 1c gene result, a hemizygous *G6PD* deficiency haplotype, was detected in two individuals (0.7%).

Class 1d gene results are heterozygous for an autosomal or X-linked recessive variant and predict, at minimum, a carrier for that recessive condition. However, the classification as having a health risk (Class 1) is made because some increased risk for specific illnesses related to the underlying genetic pathology is reported to occur in heterozygotes at frequencies greater than the general population. This is usually an increased risk for a partial or mild form of the recessive condition that may present later in life. Forty-nine Class 1d gene results were found among 48 tested individuals (16%), one individual having two such gene results (Table 3).

Class 1e gene results have two dominant variants identified in the same gene. We did not observe any individuals with a Class 1e gene result.

Class 1f gene results predict potential classical recessive disease risk. Two P/LP variants are identified in the same gene and are, or can be presumed to be, in *trans*. Because some variants may result in incomplete loss of function or modify other variants whereas other variants have more profound effects on the gene product’s function, different combinations of alleles can result in a spectrum of predicted impact and penetrance among individuals with gene results in this class. This includes allele combinations with low risk, moderate risk, and high risk. In some conditions, the risk is modified by environmental exposures, physiological differences among the sexes, and comorbidities. The gene result concept plays an important role here. Bi-allelic recessive gene results that are considered non-penetrant are not included in Class 1f (see Class 2c below).

We included gene results in this group if they conferred any reasonable level of risk because mild expression of genetic disease is often missed or misdiagnosed in routine medical care, resulting in patients unable to access disorder-specific treatments, screenings, and prevention interventions.

Table 4 shows that the gene results classified as 1f included those predicting potential atypical cystic fibrosis and male infertility, risk for clinical Hereditary Hemochromatosis type 1 or Alpha-1 Antitrypsin Deficiency, and risk for reduced penetrance *WNT10A*-related recessive ectodermal dysplasia. Seventeen individuals (5.7%) have Class 1f gene results. Excluding the *HFE* p.His63Asp homozygous results which show low penetrance, ten individuals (3.3%) have plausible health risks from a genotype consistent with recessive disease.

Class 1g are gene results that might originally be classified as 1a, but upon further investigation are determined to represent acquired somatic variants in the sampled tissue, such as blood, with sufficient positive selection to be represented at allele fractions consistent with germline variation. We identified one Class 1g gene variant. This was ultimately a secondary finding for this test revealing an asymptomatic clonal hematologic neoplasm (manuscript in preparation).

#### 3.3.2. Class 2—Carrier of a Recessive Genetic Disorder, No Known Personal Health Risk

Class 2 comprises recessive carrier gene results; that is, gene results whose effects are recessive: a single variant (2a) or two or more variants in *cis* (2b, a haplotype) plus an apparently normal gene copy are present. Class 2 gene results may occur for both autosomal and X-linked genes, the latter in individuals with two or more X chromosomes.

Five hundred and four Class 2a gene results were identified among 245 individuals. Three Class 2b gene results were identified among 3 individuals. A total of 245 individuals (81.7%) have gene results consistent with simple carrier status, i.e., Class 2a/b. The count of individuals with at least one of Class 2a, Class 2b, or the reproductively similar Class 1d autosomal recessive carrier gene results is 254 (84.7%).

Class 2c represents two variants in *trans* where the gene result, though bi-allelic, does not cause disease. While most such combinations result in recessive disease risk (Class 1f), some variants produce a mild effect on protein function such that they only lead to disease when paired with a more functionally impaired variant. Pairing with the same or another mild variant is not sufficient to cause disease and results in an unaffected, double-carrier state. The one Class 2c individual we observed is homozygous for the mild *SERPINA1* p.Glu288Val (S) allele, a genotype considered non-penetrant for clinical Alpha-1 Antitrypsin Deficiency.

Many individuals have two or more Class 2 gene results.

#### 3.3.3. Class 3—Carrier, Health Risk Uncertain

Class 3 gene results arise from technical limitations leading to uncertainty in differentiating a potential recessive disease in the individual from a carrier risk with implications only for their children. Class 3a gene results have two or more recessive variants, but the allelic phase could not be determined by the test. These cannot distinguish a recessive bi-allelic disease or disease risk (variants in *trans*) from a haplotype of variants in *cis*. The former possibility would mean a personal health risk, like Class 1f, while the latter would mean a recessive carrier, like Class 2b. We provide guidance in our messaging about the possibilities and acknowledge the uncertainty when Class 3 gene results are reported. Three individuals (1%) each had one Class 3 gene result. These occurred in the genes *ASS1, CYP21A2,* and *DNAH5*.

### 3.4. Overall Frequency of Gene Results with Personal Health Risk and Reproductive Risk Implications

Of the 300 tested patients, 103 (34.3%) have a result suggesting one or more specific personal health risks (Class 1). 245 individuals (81.7%) are a simple carrier or double carrier for one or more strictly recessive disorders (Class 2). However, Class 1d also predict recessive carrier and 1f double carrier status. Furthermore, a Class 3a result means at least a carrier and potentially predict a double carrier status. Thus, adding individuals with Class 1d, 1f, and 3a to the Class 2 gene results identifies a total of 259 individuals (86.3%) who are, for reproductive purposes, carriers or double carriers of recessive conditions. Many have both personal and reproductive (carrier) risks. This frequency does not include some Class 1a results where the dominant disease risk allele also functions as a recessive severe disease allele, such as with *BRCA2*.

### 3.5. Comparison to the American College of Medical Genetics and Genomics (ACMG) Secondary Findings Gene Results and Center for Disease Control (CDC) Tier 1 Disorders

The current test interrogates a superset of the ACMG Secondary Findings (SF) v2.0 genes [39] but not all ACMG Secondary Findings v3.0 genes [40]. Gene results meeting the ACMG SF criteria were identified 20 times with no individual having more than one such gene result (6.7% of cohort) (Table 5). This rate is similar to the 6.3% recently reported in a family medicine study targeting ACMG SF v2.0 genes [41].

Note that the SF recommendations apply to unsought results obtained through research data capture and diagnostic testing scenarios, which are different from the intentional screening program described here. The SF committee did not recommend reporting many gene results with clinical relevance because of lower penetrance, lack of medical interventions, burden to report, and other factors. However, the SF footprint has value as a curated reference gene subset when comparing frequency of findings among population genomic screening cohorts.

The CDC Office of Public Health Genomics offers evidence tiers for genomic precision health screening [42,43]. Screening for Lynch Syndrome, Hereditary Breast and Ovarian Cancer (HBOC) and Familial Hypercholesterolemia (FH) are included in the highest evidence tier, Tier 1 [44]. The Lynch Syndrome genes sequenced in our screen are *PMS2, MSH2, MLH1, MSH6,* and *EPCAM* [45,46]. One individual has a *PMS2* frameshift result. Tier 1 HBOC genes *BRCA1* and *BRCA2* were sequenced, and four individuals have P/LP results. Genes for FH typically inferred as Tier 1 include *LDLR* and *APOB.* One individual has an *LDLR* gene result consistent with FH risk. One individual has an *APOB* gene result, but the specific variant is predicted to cause autosomal co-dominant hypobetalipoproteinemia and not FH so it was not included in the CDC Tier 1 tranche. No gene results were identified in *LDLRAP1* or *PCSK9*. Taken together, seven individuals (2.3%) have gene results in the CDC Tier 1 classification. These appear in Table 5 in bold font.

## 4. Discussion

### 4.1. Cohort Uniqueness

The germline genomic screening results presented here represent clinical interrogation of 432 genes in 300 individuals for a total of 129,600 gene sequences generated and analyzed in persons not suspected of genetic disease. This is the first time both dominant and recessive health risks and recessive disease carrier status have been screened in any-health-status adults in a non-research, primary care-based, implementation with results placed in the medical record for clinical use. We analyzed gene results from the first 300 patients tested and profiled the spectrum of potential health risks and recessive carrier states among the tested individuals.

### 4.2. Cohort Representativeness

The cohort characteristics are diverse by design. The first point of contact for test availability in our pragmatic implementation is the primary care provider. This has the potential to bias the selection toward those with greater ongoing healthcare needs, including older adults. The test offering process is guided by the program director (RW), including instruction to avoid selecting based on suspected genetic disease or the absence thereof, but who is offered and agrees to testing is ultimately up to the individual and their primary care provider. Testing was offered with no biasing selection criteria, and specifically no family history of disease criteria, and at no cost [14]. Because the ordering providers were cautioned against using this test in place of traditional indication-based genetic evaluation and because the data lack hallmarks of systematic selection bias, we believe the predominance of older adults in the cohort reflects the general adult primary care population that had access to the test; however, we cannot rule out self-selection.

Zhang et al. have previously modeled and reported cost-effectiveness of circumscribed prospective combined genomic screening in young adults who may be having children and rarely have symptoms of later-onset diseases [47]. A greater benefit accrues to younger adults due to the immediate value of recessive carrier status information, as well as greater opportunity for early screening for adult-onset disorders such as cardiomyopathy and cancer susceptibility. Our program is currently evaluating approaches to increase the proportion of younger adults who learn about the test. On the other hand, older adults who share with their adult children previously unrecognized dominant health risk information as well as recessive carrier genes they may have inherited can multiply the number of informed individuals with inherited risks resolved through cascade testing.

Importantly, the local population from which the cohort is drawn presents limited ancestral diversity. 85.7% and 90.1% selected white alone race and ethnicity in the 2020 US census in the two relevant Vermont counties, respectively. As such, our results do not reflect global diversity and should not be extrapolated to populations representative of other ancestries.

Among the patient eligibility criteria in our implementation, the only systematic potentially discriminating factor is the requirement for being an attributed life in Vermont’s accountable care organization, which means eligible patients have health insurance. While those lacking health insurance are not represented, the proportion of Vermonters having health insurance in 2021 was 97% [48], likely mitigating this unintentional bias.

### 4.3. Recessive Carrier Detection

The 86% frequency of carriers of recessive conditions is consistent with the large number of genes evaluated and their known empiric carrier frequencies. The frequency is higher still when including genes selected for dominant conditions (Class 1a/b) where select variants function as recessive disease alleles. This combined gene panel does not query all known recessive disease genes. Exome or genome sequencing may detect more rare and ancestrally unique disorders.

### 4.4. Personal Health Risk Detection

Thirty four percent of tested individuals have an elevated potential personal health risk compared to the population average attributable to monogenic variation (Class 1 gene result). These included a range from high penetrance dominant disorders in cardiovascular and cancer susceptibility genes to low penetrance thrombophilia risks, individuals bi-allelic or hemizygous for reduced penetrance or conditional recessive disorders, and carriers of recessive disease with small but above average risk for later onset conditions (Table 4). Health risk gene results predicting susceptibility to cancer and cardiovascular disorders are detailed in Appendix A, Table A1 and Table A2, respectively.

For specific gene subsets, the rates we observed are in line with those of other curated panels. The rate of well-defined health risks reported in the Invitae Genetic Health (Pro-active) gene panel portion of our test (Class 1a/b) at 16% is similar to the 15.5% rate previously reported in a 10,478 unrelated person cohort using the same gene panel [15]. 6.7% meet ACMG Secondary Findings criteria and 2.3% are for CDC Tier 1 conditions [40,42,43]. The higher overall frequency of health risks detected here is likely due to the screening of more genes encompassing a wider range of penetrance and the inclusion of personal health risks detected on the carrier screen portion of the test, including attenuated health risks in certain recessive disease carriers.

If we exclude from the personal health risk tally the less traditional mechanisms (recessive disorder carriers with increased risk for attenuated disorders [Class 1d], bi-allelic recessive genotypes with unknown phase [Class 3a] or bi-allelic recessive genotypes with very low penetrance [Class 1f: HFE p.His63Asp], a relatively mild co-dominant condition [Class 1b, hypobetalipoproteinemia], and a result resolving to a somatic variant [Class 1g]), 18.6% of patients still have a defined health risk result. This health risk detection rate exceeds that of many non-genetic health-risk screening programs, and genomic screening generally does not duplicate non-genomic screening efforts. This suggests broad genomic health risk screening might provide sufficient overall value to become a routine population screening modality.

Because many of the health risk results identified are believed to have reduced penetrance, the cohort’s aggregate potential personal health impact is certainly lower than the frequency of health risk results detected. However, the surprising frequency of health risk-associated monogenic gene results suggests that health risks with germline origin may have greater impact on population disease burdens than previously appreciated, especially when their manifestations are unrecognized. The magnitude of health impact and healthcare utilization due to genomic population health screening will need to be studied over time to understand the cumulative value and its sources.

### 4.5. Uncertainty and Value

We identified uncertainty in about 1% of cases arising from technical limitations including unresolved allelic phase and pseudogene interference. Other sources of uncertainty include reduced penetrance (dominant and recessive disorders), the dynamic nature of evidence used in variant classification, actual and perceived clinical utility and advances in interventions, the genetic characteristics of the sampled population including those of the community, age, co-morbidities, and effects arising from the pragmatic self-selection approach. These results, therefore, provide a rough estimate of the potential of clinically relevant results from screening any-health-status adults using a large gene panel. Counting negative results, i.e., no reportable variants among the 432 genes, as having personal or clinical utility (avoiding future unnecessary testing, for example), the overall information value could be higher. To validate the proposed value of genomic screening, we anticipate analyzing the health and family history status at testing, result sharing patterns, how patients and providers use gene result information, costs, and clinical outcomes in this cohort.

### 4.6. Gene Result Classification System and Action Plan Messaging

Putting genomic population screening into operation in a primary care setting poses several challenges. One is how to optimize use of positive results while avoiding inappropriate responses. We produce for each set of test results an anticipatory clinical consultation messaging document, the Genomic Medicine Action Plan (GMAP), to guide patients and primary care providers in use of the test results [14]. We formalize the concept of a *gene result*, an entity comprising the gene and all its P/LP gene variants and their zygosity, to focus on the clinical implications instead of highlighting individual variant pathogenicity. This concept is imbedded in clinical genetic reasoning and is not unique, but we found its explicit formulation informs our development of a scalable system of succinct, consistent, and reusable messaging to providers and patients.

To the same end, we devised a novel clinical classification system for gene results (Figure 1, Table 2) to facilitate streamlining message development for a wide range of disorders with diverse clinical implications and inheritance patterns as well as for categorizing differing potential health and reproductive impact combinations in the cohort. It may also provide useful structure for future outcome and impact analyses. This system accounts for some but not all nuances of health risk and inheritance and we expect it will evolve with more experience.

The GMAP does incorporate the concepts embedded in penetrance. We use the term “risk” in the GMAP because “penetrance” is understood primarily by professionals with advanced human genetics training. An extended classification system that incorporates penetrance and health impact severity may help scientists refine future research intended to illuminate areas of low understanding and to test preventative health strategies. It could also help healthcare planners prioritize efforts to operationalize genomic-guided care as part of usual healthcare.

## 5. Conclusions

Clinical screening for a wide range of hidden genetic health and reproductive risks associated with succinct action and education messaging to providers and patients is feasible and scalable in a US health system. This experience illuminates a path toward using exome or genome sequences for genetic screening. The high frequency of potential health risks identified in this cohort suggests a significant influence of inborn genetic variation on health. Detection of personal health risks arising from recessive, co-dominant, and risks-to-carrier mechanisms illustrates that screening restricted to dominant conditions may leave significant gaps. This data provides general population frequencies that may be used to model health and healthcare impacts in younger individuals and couples drawn from the same population. Longitudinal study of health outcomes is still needed. Variability in penetrance underscores what we can yet discover about genetic, epigenetic, and environmental modifiers and the potential to provide more precise risk stratification as well as to stimulate new low risk interventions to modify genetic risk outcomes.

## Figures and Tables

**Table 1:** Genes evaluated, by frequency of patients having at least one P or LP variant.

Count of Patients with		
At Least 1 Variant	More Than 1 Variant or Hemizygous	Gene(s) ^1^
106	14	***HFE***
41	2	***SERPINA1***
26	1	*CFTR*
24		*BTD*
22	4	*CYP21A2*
21		*GJB2*
18		*GALT*
14		* **F5** *
13		*DHCR7*
12	1	*WNT10A*
10		*GAA*
9		*SMN1*
8		***MUTYH*** *PMM2 USH2A*
6		*HBA1 PAH PKHD1*
5		*ACSF3 **ATP7B CHEK2 F2***
4		*ACADM **BRCA2** CDH23 EYS G6PC HOGA1 MEFV RMRP SLC22A5 SLC26A4 USH1C*
3	1 (*DNAH5*)	*ALDOB **ATM** BBS1 BBS10 BLM **CACNA1S** CBS CPT2 DNAH5 F11 **FH** GBE1 GLDC **MITF** PEX1 PEX7 PYGM RAPSN SLC37A4*
2	2 (*G6PD*)	*ABCC8 AGXT ALPL ASPA **BRIP1** CAPN3 CNGB3 COL4A3 CRB1 CTNS DHDDS **DSG2** FAH G6PD GNPTAB GRHPR HBA2 HBB HEXA HGD HPS3 MKS1 **MYBPC3** MYO7A NEB PCDH15 PPT1 **SERPINC1** SGCB SMARCAL1 SMPD1 TPP1 TYMP*
1	1 (*ASS1*)	*ABC11 ACADVL ACAT1 AIRE ALMS1 AMT **APOB** ARSA ASL ASS1 ATP6V1B1 BCKDHA **BRCA1 CAV3** CERKL CHRNE CLN3 CLN5 COL4A4 COL7A1 CYBA DBT **DMD** DNAI1 DNAI2 **DSC2** DYSF ERCC6 ETFDH FANCA FANCG FKRP FKTN **FLNC** GALC GBA GCDH GLE1 HADHA **HJV HOXB13** IDUA LAMB3 **LDLR** LIPA **LMNA** LPL LRPPRC MAN2B1 MCCC2 MCOLN1 MED17 MLC1 MMAA MMACHC MPL MPV17 **MSH3** MTHFR MUT **MYH7 NBN NF1** NR2E3 **NTHL1** OAT PEX12 PEX6 PFKM **PKP2 PMS2** POMGNT1 **PRKAR1A** RPGRIP1L SACS **SDHD** SGCG SGSH SLC12A3 SLC25A15 SLC7A7 TGM1 TMEM216 **TP53** TRMU VPS13B XPA*
0	0	*ABCD1 ACAD9 ACOX1 **ACTA2 ACTC1 ACTN2 ACVRL1** ADA ADAMTS2 ADGRG1 AGA AGL AGPS ALDH3A2 ALG6 **APC** AQP2 ARG1 ARSB ASNS ATP7A ATRX **AXIN2 BAG3 BAP1 BARD1** BBS12 BBS2 BCKDHB BCS1L **BMPR1A BMPR2** BSND **CACNA1C CACNB2 CALM1 CALM2 CALM3 CASQ2 CAV1 CDC73 CDH1 CDK4 CDKN2A** CEP290 CHM CIITA CLN6 CLN8 CLRN1 COL27A1 **COL3A1** COL4A5 CPS1 CPT1A **CRYAB CSRP3** CTSK CYBB CYP11B1 CYP11B2 CYP17A1 CYP19A1 CYP27A1 DCLRE1C **DES DICER1** DLD **DSP** EDA EIF2B5 ELP1 **EMD ENG EPCAM** ERCC8 ESCO2 ETFA ETHE1 EVC EVC2 **F9** FAM161A FANCC **FBN1 FHL1 FLCN** FMR1 GALK1 GAMT **GDF2** GFM1 GJB1 **GLA** GLB1 GNE GNPTG GNS GP1BA GP9 **GPD1L GREM1 HAMP** HAX1 **HCN4** HEXB HGSNAT HLCS HMGCL HPS1 HSD17B4 HSD3B2 HYAL1 HYLS1 IDS IL2RG IVD **JUP KCNE1 KCNE2 KCNH2** KCNJ11 **KCNJ2 KCNQ1 KIT** LAMA2 LAMA3 LAMC2 **LAMP2** LCA5 **LDLRAP1** LHX3 LIFR LOXHD1 **MAX** MCCC1 **MEN1** MESP2 **MET** MFSD8 **MLH1** MMAB MMADHC MPI **MSH2 MSH6** MTM1 MTRR MTTP **MYH11 MYL2 MYL3 MYLK** NAGLU NAGS NDRG1 NDUFAF5 NDUFS6 **NF2 NKX2-5** NPC1 NPC2 NPHS1 NPHS2 NTRK1 OPA3 **OTC PALB2** PC PCCA PCCB **PCSK9 PDGFRA** PDHA1 PDHB PEX10 PEX2 PHGDH **PLN POLD1 POLE PRKAG2 PRKG1 PROC** PROP1 **PROS1** PRPS1 PSAP **PTCH1 PTEN** PTS PUS1 RAB23 **RAD51C RAD51D** RAG2 RARS2 **RB1 RBM20** RDH12 **RET** RPE65 RS1 RTEL1 **RYR1 RYR2** SAMHD1 **SCN5A SDHA SDHAF2 SDHB SDHC** SEPSECS SGCA **SGCD** SLC12A6 SLC17A5 SLC25A13 SLC26A2 SLC35A3 SLC39A4 **SLC40A1** SLC4A11 SLC6A8 **SMAD3 SMAD4 SMARCA4 SMARCB1** STAR **STK11** SUMF1 TAT **TCAP** TCIRG1 TECPR2 **TFR2 TGFB2 TGFB3 TGFBR1 TGFBR2** TH **TMEM127 TMEM43 TNNC1 TNNI3 TNNT2 TPM1 TSC1 TSC2** TSFM TTPA **VCL VHL** VPS13A VPS45 VRK1 VSX2 **WT1** XPC ZFYVE26*

**^1^** Invitae Genetic Health Screen (Pro-active) panel genes are in bold font; Invitae Comprehensive Carrier panel genes are in non-bold font; genes found on both panels are bold and underlined.

**Table 2:** Gene result clinical classes among 300 patients and predicted risks to offspring by class.

Clinical Class	Class Definition	Gene-Result Count (Unique)	Patients ^3^ (%)	Allele Transmission Frequency	Offspring Health Risk Frequency
*Negative result (Class zero)*			*n = 33 (11.0)*		
0	no P/LP variants in 432 genes	n/a	33 (11.0)	n/a	n/a
*Class 1. Health risk*		*122 (59)*	*n = 103 (34.3)*		
1a	dominant condition, one variant, targeted disease	51 (28)	47 (15.7)	0.5	0.5
1b	dominant condition, one variant, not targeted disease	1 (1)	1 (0.3)	0.5	0.5
1c	recessive condition, X-linked hemizygous variant ^1^	2 (1)	2 (0.7)	0.5	0.25
1d	attenuated risk in recessive carrier (or co-dominant)	49 (21)	48 (16.0)	0.5	0.5
1e	dominant condition, ≥2 variants in one gene	0 (0)	0 (0)	0.5–1	0.5–1
1f	recessive condition, homo- or compound heterozygous	17 (6)	17 (5.7)	1	carrier frequency/2
1g	somatic condition (variant not germline)	1 (1)	1 (0.3)	0	0
*Class 2. Carrier result, no known health risk*		*508 (246)*	*n = 245 (81.7)*		
2a	carrier, single variant	504 (244)	245 (81.7)	0.5	carrier frequency/4
2b	carrier, haplotype (two or more variants in *cis*)	3 (2)	3 (1.0)	0.5	carrier frequency/4
2c	double carrier, two *trans* variants, non-penetrant genotype	1 (1) ^2^	1 (0.3)	1	carrier frequency/2
*Class 3. Carrier, health risk uncertain*		*3 (3)*	*n = 3 (1.0)*		
3a	≥2 recessive variants in one gene, phase unknown	3 (3)	3 (1.0)	0.5–1	carrier frequency/4 to carrier frequency/2
***Total***		***632 (308)***	***n = 300***		

^1^ Single variant or haplotype in linkage disequilibrium. ^2^ Homozygous *SERPINA1* Glu288Val. ^3^ Each patient may have zero, one, or more gene results in one or more classes. n/a—not applicable.

**Table 3:** Class 1d gene results (attenuated health risks in recessive disease carriers).

Gene	Increased Health Risk in Heterozygote Recessive Disease Carriers	Patient Count
*ATM*	Cancers [18,19,20]	3
*CFTR* ^1^	Chronic pancreatitis [21,22,23]	12
*COL4A3, COL4A4*	Microscopic hematuria, age-related proteinuria, and chronic kidney disease [24,25,26]	3
*DMD*	Late-onset cardiomyopathy [27,28]	1
*GBA*	Parkinson disease, Lewy body dementia, or rapid eye movement sleep behavior disorders [29,30]	1
*HADHA*	Fatty liver of pregnancy or HELLP syndrome if carrying an affected fetus [31]	1
*HBB*	Vaso-occlusive events with extreme physical exertion, dehydration, and/or altitude [32]	1
*MEFV*	Attenuated pain syndrome [33]	4
*SERPINA1* ^2^	Decreased lung function and susceptible to lung irritants [34]	12
*WNT10A*	Mild ectodermal dysplasia and Isolated tooth agenesis [35,36,37,38]	11

^1^ c.1210-34TG [11] intronic alleles are excluded and classified 2a. ^2^ includes Z, I, and Null-Cardiff alleles without a second P/LP allele; S allele simple heterozygous gene result is assigned Class 2a.

**Table 4:** Class 1f gene results (potential health risk due to having two recessive disease alleles).

Gene	Result	Patient Count
*CFTR*	p.Phe508del and c.1210-34TG[11]T[5] (intronic)	1
*HFE*	p.Cys282Tyr (homozygous)	3
*HFE*	p.Cys282Tyr and p.His63Asp (compound heterozygous)	4
*HFE*	p.His63Asp (homozygous) ^1^	7
*SERPINA1*	p.Glu366Lys (Z allele) and p.Glu288Val (S allele)	1
*WNT10A*	p.Phe228Ile (homozygous)	1

^1^ low penetrance.

**Table 5:** ACMG Secondary Findings v2.0/v3.0 (partial), and CDC Tier 1 conditions in this cohort.

Gene	Result
***BRCA1***	p.Tyr1853*
***BRCA2***	p.Gln1408*
***BRCA2***	p.Gln2859*
***BRCA2***	p.Glu2846Glyfs*22
***BRCA2***	p.Ile2588Phefs*60
*DSC2*	c.631-2A>G (Splice acceptor)
*DSG2*	c.523+1G>A (Splice donor)
*DSG2*	p.Glu1020Alafs*18
*HFE*	p.Cys282Tyr (homozygous)
*HFE*	p.Cys282Tyr (homozygous)
*HFE*	p.Cys282Tyr (homozygous)
***LDLR***	**c.2547+1G>A (Splice donor)**
*LMNA*	p.Thr655Asnfs*49
*MYBPC3*	c.3628-41_3628-17del (Intronic)
*MYBPC3*	p.Glu542Gln (missense and splice)
*MYH7*	p.Lys865Arg
*PKP2*	p.Thr50Serfs*61
***PMS2***	p.Lys706Serfs*19
*SDHD*	p.Pro81Leu
*TP53*	p.Pro151Arg (possibly mosaic)

**Bolded** gene results predict CDC Tier 1 conditions.

## Data Availability

All sharable data is included in the manuscript, the Appendix A, and online supporting materials.

## Supplementary Materials

The following supporting information can be downloaded at: https://www.mdpi.com/article/10.3390/jpm12121962/s1, Table S1: All gene results, by clinical class.

## Appendix A. Cancer and Cardiovascular Risk Variant Tables

**Table A1:** Reported variants in genes associated with cancer risk.

Gene	Individuals (*n* = 300)	Variants
***ATM***	3	p.Glu522Ilefs*43, p.Arg2034*, p.Leu2463fs (c.7388_7389ins?)
***BRCA1***	1	p.Tyr1853*
***BRCA2***	4	p.Gln1408*, p.Ile2588Phefs*60, p.Glu2846Glyfs*22, p.Gln2859*
***BRIP1***	2	p.Arg798*, Ser895*
***CHEK2***	5	p.Ile157Thr (*n* = 3), p.Thr367Metfs*15 (*n* = 2)
***FH* ^1^**	3	p.Lys477dup
***HOXB13***	1	p.Gly84Glu
***MITF***	3	p.Glu318Lys
***MSH3* ^2^**	1	deletion (exon 16)
***MUTYH* ^2^**	8	p.Tyr179Cys (*n* = 3), p.Arg274Gln (*n* = 2), p.Gly396Asp (*n* = 4),
***NBN***	1	p.Lys233Serfs*5
***NF1***	1	p.Leu1183Arg (possibly mosaic)
***NTHL1* ^2^**	1	p.Gln90*
***PMS2***	1	p.Lys706Serfs*19
***PRKAR1A***	1	c.709-7_709-2del
***SDHD***	1	p.Pro81Leu
***TP53***	1	p.Pro151Arg (possibly mosaic)

^1^ Pathogenic variant for a recessive metabolic disorder, Fumarase Deficiency; no cancer risk for this variant [49]. ^2^ Principally a recessive cancer risk gene. All individuals were heterozygous for the indicated variants. No reportable variants were detected among the following cancer risk genes: *APC, AXIN2, BAP1, BARD1, BMPR1A, CDC73, CDH1, CDK4, CDKN2A, DICER1, EPCAM, FLCN, GREM1, KIT, MAX, MEN1, MET, MLH1, MSH2, MSH6, NF2, PALB2, PDGFRA, POLD1, POLE, PTCH1, PTEN, RAD51C, RAD51D, RB1, RET, SDHA, SDHAF2, SDHB, SDHC, SMAD4, SMARCA4, SMARCB1, STK11, TMEM127, TSC1, TSC2, VHL, WT1*.

**Table A2:** Reported variants in genes associated with cardiovascular risk.

Gene	Individuals (*n* = 300)	Variants
***APOB* ^1^**	1	p.Phe2656Thrfs*10
***CAV3***	1	p.Ala93Thr
***DMD* ^2^**	1	p.Val2159Serfs*4
***DSC2***	1	c.631-2A>G (splice acceptor)
***DSG2***	2	c.523+1G>A (splice donor), p.Glu1020Alafs*18
***F2* ^3^**	5	c.*97G>A (also known as c.20210G>A)
***F5***	14	p.Arg534Gln (also known as “Leiden”)
***FLNC* ^4^**	1	deleted gene
***LDLR***	1	c.2547+1G>A (splice donor)
***LMNA***	1	p.Thr655Asnfs*49
***MYBPC3***	2	c.3628-41_3628-17del (Intronic), p.Glu542Gln (missense and splice)
***MYH7***	1	p.Lys865Arg
***PKP2***	1	p.Thr50Serfs*61
***SERPINC1* ^3^**	2	p.Pro73Leu

^1^ Pathogenic, but not for the targeted phenotype (FH). ^2^ Female heterozygote for X-linked variant. ^3^ One individual with clotting variants in two genes. ^4^ Determined to be somatic hematologic loss, not germline. All individuals were heterozygous for the indicated variants. No reportable variants were detected among the following cardiovascular risk genes: *ACTA2, ACTC1, ACTN2, ACVRL1, BAG3, BMPR2, CACNA1C, CACNB2, CALM1, CALM2, CALM3, CASQ2, CAV1, COL3A1, CRYAB, CSRP3, DES, DSP, EMD, ENG, F9, FBN1, FHL1, GDF2, GLA, GPD1L, HCN4, JUP, KCNE1, KCNE2, KCNH2, KCNJ2, KCNQ1, LAMP2, LDLRAP1, MYH11, MYL2, MYL3, MYLK, NKX2-5, PCSK9, PLN, PRKAG2, PRKG1, PROC, PROS1, RBM20, RYR2, SCN5A, SGCD, SMAD3, SMAD4, TCAP, TGFB2, TGFB3, TGFBR1, TGFBR2, TMEM43, TNNC1, TNNI3, TNNT2, TPM1, VCL*.
