# Supplementary material for: Real-World Results from Combined Screening for Monogenic Genomic Health Risks and Reproductive Risks in 300 Adults
Source: J Pers Med. 2022 Nov 28;12(12):1962. doi: 10.3390/jpm12121962 (PMC9782229; doi:10.3390/jpm12121962)
Supplement: Supplementary file 1 [file jpm-12-01962-s001.zip › jpm-1983119-supplementary.pdf]

Supplemental Table S1: All gene results, by clinical class.

Gene	Result	Count
	**Class Ia**	**51**
BRCA1	p.Tyr1853*	1
BRCA2	p.Gln1408*	1
BRCA2	p.Gln2859*	1
BRCA2	p.Glu2846Glyfs*22	1
BRCA2	p.Ile2588Phefs*60	1
BRIP1	p.Arg798*	1
BRIP1	p.Ser895*	1
CHEK2	p.Ile157Thr	3
CHEK2	p.Thr367Metfs*15	2
DSC2	c.631-2A>G (SA)	1
DSG2	c.523+1G>A (SD)	1
DSG2	p.Glu1020Alafs*18	1
F2	c.*97G>A (c.20210G>A)	5
F5	p.Arg534Gln (Leiden)	14
HOXB13	p.Gly84Glu	1
LDLR	c.2547+1G>A (SD)	1
LMNA	p.Thr655Asnfs*49	1
MITF	p.Glu318Lys	3
MYBPC3	c.3628-41_3628-17del (I)	1
MYBPC3	p.Glu542Gln (missense and splice)	1
MYH7	p.Lys865Arg	1
NF1	p.Leu1183Arg (mosaic?)	1
PKP2	p.Thr50Serfs*61	1
PMS2	p.Lys706Serfs*19	1
PRKAR1A	c.709-7_709-2del	1
SDHD	p.Pro81Leu	1
SERPINC1	p.Pro73Leu	2
TP53	p.Pro151Arg (mosaic?)	1
	**Class Ib**	**1**
APOB	p.Phe2656Thrfs*10	1
	**Class Ic**	**2**
G6PD	p.[Val68Met Asn126Asp] (A- haplotype)	2
	**Class Id**	**49**
ATM	p.Arg2034*	1
ATM	p.Glu522Ilefs*43	1
ATM	p.Leu2463fs	1
CFTR	c.1210-34TG[12]T[5] (I)	5
CFTR	p.Asp1152His	1
CFTR	p.Leu206Trp	1
CFTR	p.Phe508del	5
COL4A3	p.Gln10*	1
COL4A3	p.Gly395Glu	1
COL4A4	c.1696+1G>T	1
DMD	p.Val2159Serfs*4	1
GBA	p.Asn409Ser	1
HADHA	p.Glu641Serfs*12	1
HBB	p.Glu7Val (HbS)	1
MEFV	p.Ala744Ser	1
MEFV	p.Val726Ala	3
SERPINA1	p.Arg63Cys (I allele)	2
SERPINA1	p.Asp280Val (Null Cardiff allele)	1
SERPINA1	p.Glu366Lys (Z allele)	9
WNT10A	p.Cys107*	2
WNT10A	p.Phe228Ile (LP)	9
	**Class If**	**17**
CFTR	p.Phe508del and c.1210-34TG[11]T[5] (I) (bi-allelic)	1
HFE	p.Cys282Tyr (homozygous)	3
HFE	p.Cys282Tyr and p.His63Asp (bi-allelic)	4
HFE	p.His63Asp (homozygous) (LP)	7
SERPINA1	p.Glu366Lys (Z allele) and p.Glu288Val (S allele) (bi-allelic)	1
WNT10A	p.Phe228Ile (homozygous) (LP)	1
	**Class Ig**	**1**
FLNC	deleted gene	1
	**Class IIa**	**504**
ABCB11	p.Arg487His	1
ABCC8	c.3989-9G>A (I)	1
ABCC8	p.Gly1400Arg	1
ACADM	p.Lys329Glu	3
ACADM	p.Tyr337Ser	1
ACADVL	p.Glu277del	1
ACAT1	p.Gly183Arg	1
ACSF3	p.Arg558Trp	4
ACSF3	p.Glu359Lys	1
AGXT	p.Phe152Ile	1
AGXT	p.Trp108Arg	1
AIRE	p.Leu323Serfs*51	1
ALDOB	p.Ala150Pro	3
ALMS1	p.Val3906Glyfs*2	1
ALPL	p.Arg391Valfs*12	1
ALPL	p.Phe327Cys	1
AMT	c.259-2A>C	1
ARSA	p.Ile181Ser	1
ASL	p.Arg12Gln	1
ASPA	p.Ala305Glu	1
ASPA	p.Glu285Ala	1
ATP6V1B1	p.Leu12Serfs*8	1
ATP7B	p.Arg1319*	1
ATP7B	p.Asp765Asn	1
ATP7B	p.Gly869Arg	2
ATP7B	p.Thr991Met	1
BBS1	p.Met390Arg	3
BBS10	p.Cys91Leufs*5	2
BBS10	p.Tyr559*	1
BCKDHA	p.Arg117Cys (LP)	1
BLM	c.3558+1G>T	1
BLM	p.Arg899*	1
BLM	p.Gln672Arg	1
BTD	p.Asp444His (PD)	23
BTD	p.Gly34Valfs*35	1
CACNA1S	p.Cys245Alafs*89	1
CACNA1S	p.Gln7*	1
CACNA1S	p.Leu1612Trpfs*22	1
CAPN3	p.Arg489Gln	1
CAPN3	p.Arg490Trp	1
CAV3	p.Ala93Thr	1
CBS	p.Ile278Thr	2
CBS	p.Pro49Leu	1
CDH23	c.7482+2T>C	1
CDH23	p.Arg1746Gln	1
CDH23	p.Leu1218Phefs*27	1
CDH23	p.Thr1587Cysfs*4	1
CERKL	p.Pro287Leufs*10	1
CFTR	c.1210-34TG[11]T[5] (I) (LP)	13
CHRNE	p.Tyr35His	1
CLN3	p.Arg405Trp	1
CLN5	p.Trp224*	1
CNGB3	p.Glu336*	1
CNGB3	p.Pro94Leufs*30	1
COL7A1	p.Gly2177Trpfs*113	1
CPT2	p.Ser113Leu	3
CRB1	p.Cys948Tyr	1
CRB1	p.Ile167_Gly169del	1
CTNS	c.970+5G>A (I)	1
CTNS	p.Gly169Asp	1
CYBA	p.Arg90Trp	1
CYP21A2	c.293-13C>G (I)	2
CYP21A2	p.Arg357Trp	1
CYP21A2	p.Gln319*	2
CYP21A2	p.Ile173Asn	1
CYP21A2	p.Pro454Ser	6
CYP21A2	p.Val282Leu	6
DBT	p.Arg301Cys	1
DHCR7	c.964-1G>C (SA)	11
DHCR7	p.Trp151*	1
DHCR7	p.Val326Leu	1
DHDDS	c.441-24A>G (I)	2
DNAH5	exon 57-59 deletion	1
DNAH5	p.Arg4127His	1
DNAI1	c.48+2dup (I)	1
DNAI2	p.Asp597Valfs*8	1
DYSF	c.1053+1G>A	1
ERCC6	p.Arg1087*	1
ETFDH	p.Leu334Pro	1
EYS	c.2137+1G>A (SD)	1
EYS	p.Gln171*	1
EYS	p.His263Thrfs*15	1
EYS	p.Thr2805Asnfs*7	1
F11	p.Cys146*	1
F11	p.Glu135*	1
F11	p.Glu19Asnfs*3	1
FAH	c.1062+5G>A (I)	1
FAH	c.554-1G>T (SD)	1
FANCA	deletion (exons 11-29)	1
FANCG	c.1480+1G>C (SD)	1
FH	p.Lys477dup	3
FKRP	p.Leu276Ile	1
FKTN	p.Cys137*	1
G6PC	p.Arg83Cys	1
G6PC	p.Gln27Argfs*9	1
G6PC	p.Gln319*	1
G6PC	p.Gln347*	1
GAA	c.-32-13T>G (I)	3
GAA	p.Arg281Gln	1
GAA	p.Arg281Trp	1
GAA	p.Asp404Asn	1
GAA	p.Asp459His	1
GAA	p.Gly293Arg	1
GAA	p.Met519Val	1
GAA	p.Trp746Cys	1
GALC	p.Ser303Phe	1
GALT	c.-119_-116del (NC) (Duarte)	17
GALT	deleted gene	1
GBE1	c.691+2T>C	1
GBE1	p.Thr254Ala	1

Supplemental Table S1: All gene results, by clinical class.

GBE1	p.Tyr329Cys	1
GCDH	p.Met191Thr	1
GJB2	p.Gly12Valfs*2	4
GJB2	p.Leu56Argfs*26	2
GJB2	p.Leu90Pro	2
GJB2	p.Met34Thr	12
GJB2	p.Val37Ala	1
GLDC	deletion (exons 1-15)	1
GLDC	p.Arg739His	1
GLDC	p.Pro345Thr	1
GLE1	p.Arg569His	1
GNPTAB	p.Leu1168Glnfs*5	2
GRHPR	p.Asp35Thrfs*11	2
HBA1	CNV x4	1
HBA1	deleted gene	5
HBA2	deleted gene	2
HBB	p.Gly70Ser	1
HEXA	c.1073+1G>A	1
HEXA	c.805+1G>A	1
HFE	p.Cys282Tyr	23
HFE	p.His63Asp (LP)	69
HGD	p.Gly161Arg	1
HGD	p.Met368Val	1
HJV	p.Gly302Val	1
HOGA1	c.700+5G>T (I)	3
HOGA1	p.Ala36Val	1
HPS3	p.Arg397Trp	1
HPS3	p.Gly146*	1
IDUA	c.386-2A>G	1
LAMB3	p.Val948Cysfs*82	1
LIPA	p.Ser133*	1
LPL	p.Arg197Cys	1
LRPPRC	p.Ala354Val	1
MAN2B1	p.Trp714Arg	1
MCCC2	p.Gly475Arg	1
MCOLN1	p.Thr232Pro	1
MED17	p.Gln482*	1
MKS1	p.Cys492Trp	1
MKS1	p.Gln408*	1
MLC1	deletion (exon 3)	1
MMAA	p.Arg145*	1
MMACHC	p.Arg91Lysfs*14	1
MPL	p.Lys553Argfs*77	1
MPV17	p.Pro98Leu	1
MSH3	deletion (exon 16)	1
MTHFR	p.Arg52Gln	1
MUT	p.Ala676Thr	1
MUTYH	p.Arg274Gln	2
MUTYH	p.Gly396Asp	4
MUTYH	p.Tyr179Cys	2
MYO7A	p.Arg1240Gln	1
MYO7A	p.Arg634*	1
NBN	p.Lys233Serfs*5	1
NEB	c.12331-1G>A (SA)	1
NEB	p.Trp3603*	1
NR2E3	c.119-2A>C (SA)	1
NTHL1	p.Gln90*	1
OAT	p.Arg398*	1
PAH	c.1315+1G>A	1
PAH	p.Arg408Trp	2
PAH	p.Glu280Lys	1
PAH	p.Pro281Leu	1
PAH	p.Val230Ile	1
PCDH15	p.Arg1106*	1
PCDH15	p.Arg245*	1
PEX1	p.Arg949Gln	1
PEX1	p.Ile700Tyrfs*42	2
PEX12	p.Tyr74*	1
PEX6	p.Leu614Argfs*5	1
PEX7	p.Gly217Arg	1
PEX7	p.Leu292*	2
PFKM	c.237+1G>A (SD)	1
PKHD1	c.390+1G>T (SD)	1
PKHD1	p.Ala1254Glyfs*49	1
PKHD1	p.Arg494*	1
PKHD1	p.Ile2331Lys	2
PKHD1	p.Thr36Met	1
PMM2	c.255+2T>C (SD)	1
PMM2	p.Arg141His	6
PMM2	p.Val67Gly	1
POMGNT1	c.1539+1G>A (SD)	1
PPT1	p.Arg151*	2
PYGM	p.Arg50*	1
PYGM	p.Lys754Asnfs*49	1
PYGM	p.Phe599Leufs*6	1
RAPSN	p.Asn88Lys	3
RMRP	n.-17_-2dup (NC, promoter)	1
RMRP	n.-25_-3dup (NC)	1
RMRP	n.71A>G (RNA change)	1
RMRP	NR_003051.3:n.239C>T (RNA Change)	1
RPGRIP1L	p.Arg1177*	1
SACS	deleted gene	1
SERPINA1	p.Glu288Val (S allele) (LP)	27
SGCB	p.Ser114Phe	2
SGCG	Deleted gene	1
SGSH	p.Met1?	1
SLC12A3	c.506-1G>A	1
SLC22A5	c.-149G>A (NC)	2
SLC22A5	p.Ala214Val	1
SLC22A5	p.Pro46Ser	1
SLC25A15	p.Glu180Lys	1
SLC26A4	c.1001+1G>A (SD)	1
SLC26A4	p.Glu384Gly	1
SLC26A4	p.Gly209Val	2
SLC37A4	p.Ala113Glyfs*18	1
SLC37A4	p.Leu116Glyfs*31	1
SLC37A4	p.Trp96*	1
SLC7A7	deletion (exons 4-5)	1
SMARCAL1	p.Glu848*	1
SMARCAL1	p.Phe279Ser	1
SMN1	c.*3+80T>G (g.27134T>G) (ICR)	1
SMN1	deleted gene	8
SMPD1	p.Asp253His	1
SMPD1	p.Gly244Arg	1
TGM1	p.Trp263	1
TMEM216	p.Asn121Glufs*15	1
TPP1	p.Arg208*	1
TPP1	p.Lys18*	1
TRMU	p.Leu195Ilefs*12	1
TYMP	p.Glu289Ala	1
TYMP	p.Val208Met	1
USH1C	c.216G>A (Silent)	2
USH1C	c.748_759+5del (SD)	1
USH1C	deletion (exon 9)	1
USH2A	c.949 C>A	1
USH2A	p.Arg205Glufs*11	1
USH2A	p.Arg4192His	1
USH2A	p.Glu3562*	1
USH2A	p.Glu767Serfs*21	1
USH2A	p.Ile1183Phefs*19	1
USH2A	p.Ile5166Val	1
USH2A	p.Trp3955*	1
VPS13B	c.10942+1G>A (SD)	1
XPA	p.Gln185His	1
	**Class IIb**	**3**
CYP21A2	c.293-13C>G (I) and p.Gly111Valfs*21 and p.[Ile237Asn Val238Glu Met240Lys] and p.Ile173Asn and p.Leu308Phefs*6 and p.Pro31Leu (in cis)	1
CYP21A2	p.Gln319* and gene duplication (phase unknown)	2
	**Class IIc**	**1**
SERPINA1	p.Glu288Val (S allele) (homozygous) (LP)	1
	**Class IIIa**	**3**
ASS1	p.Val269Met and p.Gly390Arg (bi-allelic phase unknown)	1
CYP21A2	p.Val282Leu (zygosity uncertain) p.Leu308Phefs*6 p.Arg357Trp as well as p.Ile237Asn, p.Val238Glu, p.Met240Lys (phase unknown)	1
DNAH5	c.2744-1G>T and p.Arg36Glyfs*8 (bi-allelic phase unknown)	1

Abbreviations: SA – splice acceptor, SD – splice donor, I – intronic, NC – non-coding, LP – low penetrance, PD – partial deficiency, ICR – increased carrier risk
